# Diagnostic traction and dorsal locking plate stabilization of a fifth and sixth thoracic vertebral fracture/luxation in a golden retriever: Case report

**DOI:** 10.3389/fvets.2022.1011983

**Published:** 2022-12-23

**Authors:** William J. Tammaro, Peter J. Early, Robert Bergman, Brian L. Petrovsky, Karl H. Kraus

**Affiliations:** ^1^Veterinary Clinical Sciences, College of Veterinary Medicine, Iowa State University, Ames, IA, United States; ^2^Clinical Sciences, Veterinary Hospital, North Carolina State University, Raleigh, NC, United States; ^3^Veterinary Neurology and Neurosurgery, Carolina Veterinary Specialists, Rock Hill, SC, United States; ^4^Arizona Canine Orthopedics and Sports Medicine, Scottsdale, AZ, United States

**Keywords:** diagnostic traction, dorsal locking plates, spinal stabilization, thoracic vertebral fracture/luxation, case report

## Abstract

Traction was used to diagnose instability of a T5-T6 traumatic luxation that was stabilized with locking plates in the laminae and dorsal pedicles. A two-year-old, 27 kg, female spayed golden retriever was presented to a veterinary teaching hospital after being referred for possible mandibular and spinal fractures after being hit by a car. The dog presented non-ambulatory paraparetic with intact pain perception. Computed tomographic (CT) imaging showed a fifth and sixth thoracic vertebral fracture/luxation, with and without manual traction. Surgical stabilization of the spine was performed with bilateral dorsally placed locking plates (String-of-Pearls, Orthomed, UK) in the laminae and dorsal aspects of the vertebral pedicles. The dog recovered well, and neurologic status improved significantly overnight and continued to improve up until discharge, which was 6 days postoperatively. Upon recheck exam at 8 weeks postoperatively, the dog appeared neurologically normal with no obvious surgical complications. This case demonstrates that diagnostic traction—the process of pulling, during imaging, on the dog's pelvis while the forelimbs are secured in extension—demonstrated instability of the spine which was not readily apparent on initial CT imaging. Additionally, the dorsal locking plate stabilization is a viable fixation option that provided acceptable stabilization of the mid-thoracic vertebrae.

## 1. Introduction

Mid-thoracic vertebral fractures are uncommon due to the inherent rigidity of the spine in this area and often do not require surgical intervention (1). Despite the relative stability of the mid-thoracic spine, injuries can occur. Most commonly, they are due to vehicular trauma, with road traffic accidents accounting for 40–60% of vertebral fractures/luxations in dogs and cats (1). In cases where surgical intervention is indicated, there are a wide variety of techniques that have been described (2). However, some of these techniques are technically challenging to employ in the mid-thoracic spine.

To the authors' knowledge, no prior reports of diagnostic traction are being used to evaluate the spine's stability in dogs. Additionally, to the authors' knowledge, no prior reports of using dorsal locking plate stabilization of the dog mid-thoracic spine are being used. In human medicine, posterior/dorsal stabilization has been well documented and is the technique of choice for many surgeons (3).

This report's aim is to describe the technique of diagnostic traction with advanced imaging and how this can be a useful clinical tool. Additionally, this report describes the favorable outcome of dorsal locking plate stabilization of the vertebral column and how it can be used to achieve good clinical results less invasively when compared to a ventral approach or with vertebral body implants.

## 2. Case presentation

### 2.1. Clinical history

A 2-year-old 27 kg female spayed Golden Retriever was referred to the Hixon-Lied Small Animal Teaching Hospital at Iowa State University for evaluation and treatment of possible mandibular and spinal fractures after being hit by a car. Prior to referral, the patient had been in good health.

### 2.2. Physical exam

An abbreviated physical exam was performed due to the nature of the presenting injuries; however, the patient appeared stable with heart rate, respiratory rate, and temperature all within normal limits. Her gait was reported to be non-ambulatory paraparesis, but was initially not examined as she was strapped to a board prior to anesthesia and imaging. Pain around the cervical and thoracic spine was noted. Increased free fluid in the abdomen was noted and, after abdominocentesis, revealed to be blood. Neurological examination showed appropriate mentation, superficial and deep pain perception, and reflexes in all limbs. The cranial nerves were within normal limits. However, the pelvic limbs were hyperreflexic. Neurolocalization was T3-L3 myelopathy. A good prognosis for ambulation was given to the owners.

### 2.3. Diagnostic imaging

**Figure 1:**
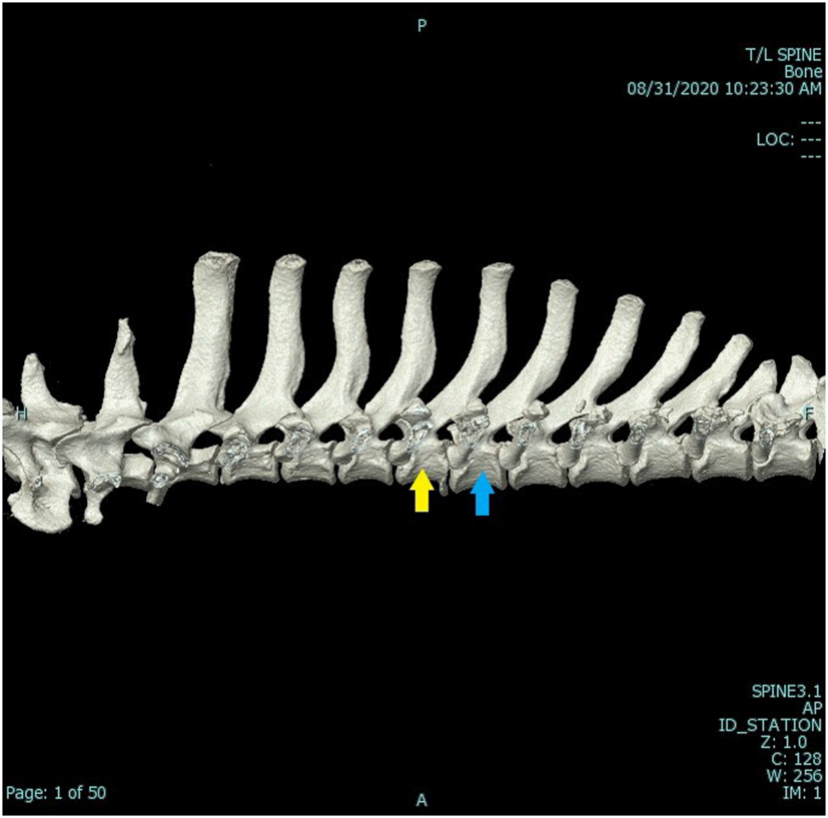
Computed tomographic (CT) 3-D reconstruction of the patient's spine with no traction applied. The yellow arrow indicates T5, and the blue arrow indicates T6. This image was taken preoperatively, and the patient was under general anesthesia.

**Figure 2:**
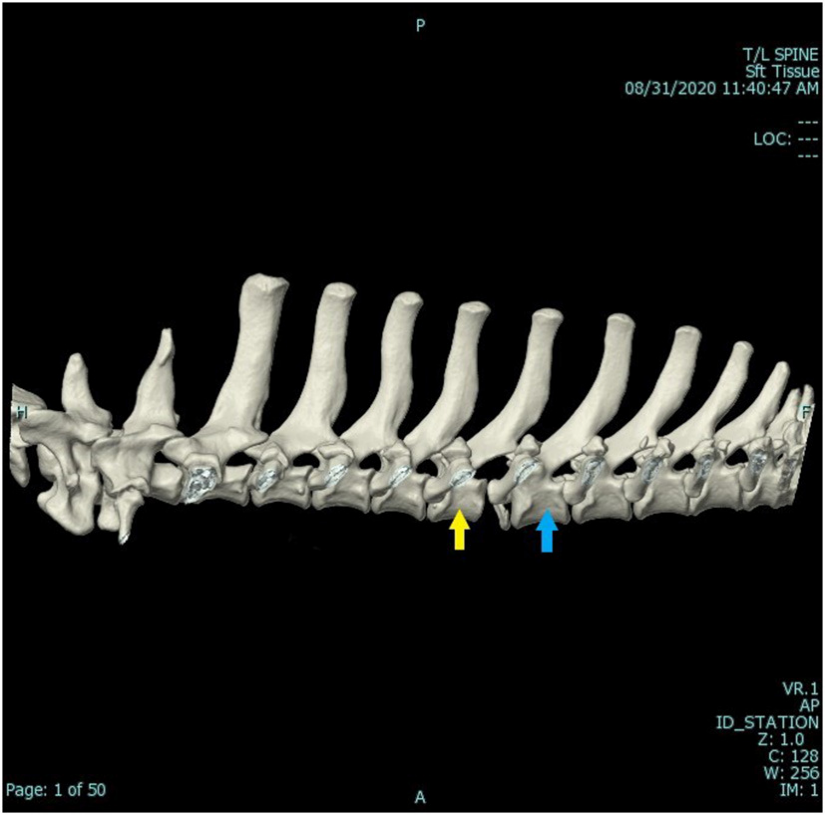
CT 3-D reconstruction of the patient's spine while traction was applied. The patient was in dorsal recumbency in the gantry of the CT. The forelimbs were held in extension and secured to the movable table. Traction, approximately enough to lift the patient (20 kg), was applied to the pelvic limbs which were then secured to the table thereby maintaining traction. The yellow arrow indicates T5, and the blue arrow indicates T6. This image was take preoperatively, and the patient was under general anesthesia.

A computed tomography (CT) scan was ordered and performed using a 16-slice Canon Aquilion scanner (TSX201A, Canon Medical Systems, Tustin, California). A three-dimensional (3-D) reconstructed image of the dog's vertebral column can be seen in Figure 1. A second CT scan was performed using traction on the spine. The patient was in dorsal recumbency in the gantry of the CT. The forelimbs were held in extension and secured to the movable table. Tape was applied to the pelvic limbs and secured to the other end of the movable table. Traction, in this case, approximately 20 kg of force, was enough to lift the patient. Therefore, the patient was in traction and personnel could leave the room, thereby mitigating radiation exposure. The 3-D reconstructed image of the spine while traction was applied can be seen in Figure 2. The CT scans, as read by a board-certified radiologist, showed multiple fractures of the thoracic vertebral column/ribs. The left first rib had a long curved oblique non-displaced fracture. The left fifth transverse process/costal fovea was dorsally and laterally displaced. The caudoventral endplate of T5 was avulsed and was affixed to the ventral aspect of the T5-T6 intervertebral disc space. The T5-T6 intervertebral disc space was markedly widened, and this was a dynamic change as shown while under traction. The spinal cord at this level contacted the left T6 pedicle. The left mammary process and transverse process of T6 were dorsally and laterally displaced, and the left costal head and tubercle were dorsally displaced with mild supination. A transverse fracture of the left transverse process of T7 was dorsally displaced, and a similar fracture was present at T8. The area of concern was the T5-T6 dynamic instability secondary to the caudoventral vertebral body fracture of T5 and soft tissue disruption. This finding was consistent with traumatic vertebral instability. Radiographs and CT imaging of the skull were also performed to confirm a mandibular fracture.

### 2.4. Surgical procedure

**Figure 3:**
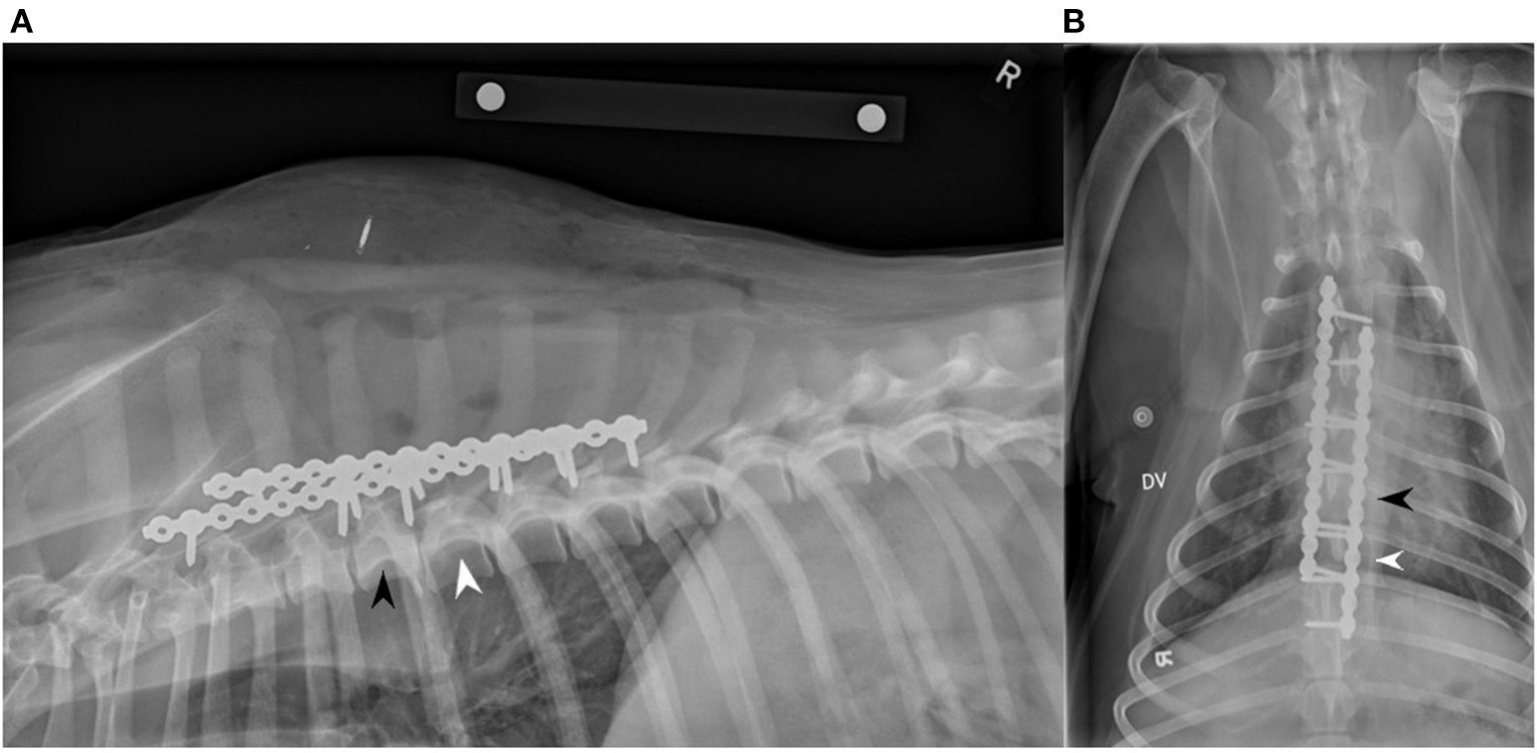
Right lateral **(A)** and dorsoventral **(B)** two view post-operative radiographs showing two SOP^®^ dorsal locking plates successfully implanted. A 14-hole SOP^®^ locking plate was used on the left side with six 2.7 mm screws placed into the bases of the dorsal spinous processes/laminae of T3, T4, T5, T6, T7, and T8. A second 14-hole SOP^®^ locking plate was used on the right side with five 2.7 mm screws placed into the bases of the dorsal spinous processes/laminae of T2, T4, T5, T6, and T7. The black arrowhead indicates T5, and the white arrowhead indicates T6.

The dog was given 270 mcg of fentanyl IV, as a pre-anesthetic medication. The dog was then induced with 54 mg of lidocaine IV, 14 mg of ketamine IV, and 14 mg of propofol IV. Once induced, the dog was maintained under gas anesthetic using isoflurane, positioned into sternal recumbency, and prepared for surgery. A 25 cm dorsal midline incision over the spinous processes of T2-T9 was made and, hemorrhage was controlled using monopolar and bipolar cautery. The dorsal lumbar fascia was exposed, and the deep thoracic fascia on either side of the dorsal spinous processes was sharply excised. Epaxial muscles were elevated from both sides of the dorsal spinous processes to the level of the articular facets using sharp and blunt dissection along with retractors and periosteal elevators. The articular facets were visualized to be in close alignment. A 14-hole String-of-Pearls (SOP^®^) locking plate was placed at the base of the spinous processes dorsal to the vertebral canal along the left lateral aspect of T3-T9. No implant contouring was assessed to be needed. A 2.5 mm drill was used in concert with a guide to place the screw holes, which were subsequently measured with a depth gauge. Six 2.7 mm cortical screws on the left plate were placed in the bases of the dorsal spinous processes of T3, T4, T5, T6, T7, and T8. The process was repeated on the right side in a similar fashion. Another 14-hole SOP^®^ locking plate was placed on the right side and five 2.7 mm cortical screws were placed in the bases of the dorsal spinous processes of T2, T4, T5, T6, and T7. The incision was routinely closed in typical three layers (fascia, subcutaneous, and skin). The total surgical time was just under 2 h. After the spinal stabilization procedure, the patient was moved into right lateral recumbency, and the left mandibular fracture was repaired using cerclage wire. Post-operative lateral and dorsoventral radiographs were performed and showed near-anatomical alignment and adequate reduction, stabilization, and placement of plates and screws, as seen in Figure 3. The patient recovered uneventfully and was transferred to the ICU for overnight monitoring. Postoperatively, the dog was given 24 mg of both propofol and ketamine IV and 0.025 mg of dexmedetomidine IV for analgesia and sedation.

### 2.5. Post-operative care

The patient recovered from surgery well and was ambulatory paraparetic the following day. Post-operative medications included carprofen (100 mg tablet, ½ tablet PO BID), gabapentin (300 mg tablet PO Q8–12 h), trazadone (100 mg tablet PO Q8–12 h), and amoxicillin and clavulanic acid (375 mg tablet PO BID). Two days postoperatively, the dog was transferred to ISU's rehabilitation service. Rehabilitation involved short elimination walks, passive range of motion, and massage. Upon discharge, 6 days postoperatively, the dog was ambulatory with no obvious ataxia. Neurologic examination at the time of discharge was considered within normal limits.

### 2.6. Recheck exam

**Figure 4:**
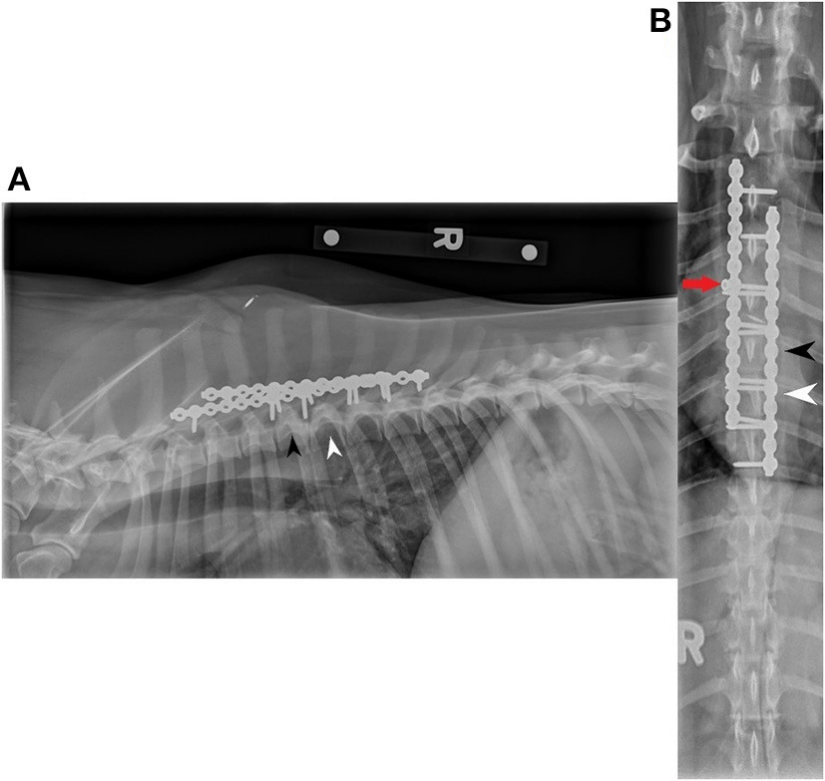
Right lateral **(A)** and dorsoventral **(B)** two view recheck radiographs, taken 8 weeks postoperatively, showing the same two SOP^®^ dorsal locking plates as in Figure 3. The black arrowhead indicates T5, and the white arrowhead indicates T6. The red arrow indicates slight loosening of the second screw in the right plate. The screw was still engaged in the vertebra, and no evidence of inappropriate radiolucent halo was seen. No other screws showed evidence of migration and the apparatus was still deemed secure.

At the 8-week recheck examination, the physical exam showed that the dog had no lameness and was comfortable with palpation of the spine. Orthopedic and neurologic examinations were unremarkable, and no obvious deficits were noted. Repeat lateral and dorsoventral radiographs were taken (Figure 4). The second cortical screw (engaged in T4) on the right side had partial screw loosening but was still engaged in the vertebra. Otherwise, the apparatus was stable. No other abnormalities (infection, migration, movement) were noted.

### 2.7. Long term follow up

Two years after presentation, the owners reported by phone that the patient was fully functional without any obvious neurologic deficits or lameness.

## 3. Discussion

### 3.1. Diagnostic traction

Mid-thoracic vertebral fractures/luxations are uncommon due to the increased musculature, ribs, and soft fibrous tissues that help to protect and stabilize this area in trauma. The thoracic spine is inherently more stable because larger longissimus thoracus muscles and a larger interspinous ligament provide added stability. Articulation of the ribs and associated intercostal muscles provide additional stability (4, 5).

The stability of the vertebral column is typically assessed in three compartments: dorsal, middle, and ventral. The dorsal compartment contains the spinous processes, vertebral laminae, cranial and caudal articular processes, vertebral pedicles, and the dorsal ligamentous complex (supraspinous ligament, interspinous ligament, joint capsule, and ligamentum flavum) (1, 2, 6, 7). The middle compartment contains the dorsal aspect of the vertebral body, the dorsal annulus fibrosus, and the dorsal longitudinal ligament (1, 2, 6, 7). The ventral compartment contains the ventral aspect of the vertebral body, the lateral and ventral annulus fibrosus, the nucleus pulposus, and the ventral longitudinal ligament (1, 2, 6, 7). If two or three of these compartments are compromised, the spine is considered unstable and surgical intervention is often indicated (2).

In this dog, the soft tissue structures could not be adequately assessed from the CT images alone. Magnetic resonance imaging (MRI) may have provided more information as it would have evaluated the soft tissue structures and associated trauma more precisely. However, this would have greatly increased anesthetic time. Also, signal changes of soft tissue structures seen on MRI images may not have correlated with structural integrity. A report has shown that complete agreement between MRI and CT is limited regarding fracture location and that mistakes are commonly made in all vertebral structures suggesting that MRI is a poor modality for assessing fracture morphology (7). In this dog, manual traction under anesthesia clearly demonstrated loss of structural integrity of the supportive structures between T5 and T6 (Figure 2).

The force that was used to distract was subjective but was enough to lift the patient. This traction was estimated to be about 20 kgs. In this case, the CT scan with traction clearly demonstrated instability, and therefore the amount of traction used was sufficient. Determining an appropriate distractive force would be difficult due to differences in the size of the patient and the degree of trauma. Additionally, the strength of the person taping the dog's limbs to the movable table to maintain the traction is another variable to be considered. In this dog, an initial interpretation by an experienced surgeon was that only the ventral compartment of the vertebral column was disrupted. However, in fact, all three compartments were disrupted, and therefore surgery was recommended.

### 3.2. Spinal stabilization

Several methods of stabilization of the vertebral column have been described. These methods include cross pins and polymethylmethacrylate (PMMA), locking plates, external fixators, vertebral body plates, modified segmental fixation, tension band stabilization, and spinous process plates (2). There have been few reports of mid-thoracic spinal stabilization in veterinary medicine, none of which describe only dorsal stabilization, to the authors' knowledge. Mathiesen et al. presented a case series that reported ventral stabilization of the spine using SOP^®^ locking plates in six pugs with thoracic kyphosis (8). Another report by Farré Mariné et al. described the surgical technique used to treat congenital thoracic vertebral body malformations using unilateral vertebral body distraction and ventral stabilization with monocortical screws and PMMA (9). Both reports described a transthoracic approach to achieve ventral stabilization of the thoracic spine impacted by congenital abnormalities (8, 9). Guiot et al. presented a case report that discussed ventral stabilization of a T5 comminuted fracture *via* median sternotomy in a small dog (10). Klatzkow and colleagues presented a case report that discussed a median sternotomy technique for ventral stabilization of a T2-T3 luxation in a dog (11). A ventral approach to the mid-thoracic spine with SOP^®^ locking plates is a highly invasive and lengthy procedure, with risks of iatrogenic damage to adjacent, vital structures. Thus, the surgeons, in this case, opted for a dorsal approach with SOP^®^ locking plates. There are several publications documenting the use of 3-D printed drill guides in spinal stabilizations of vertebral malformations (12–14). However, these take time to produce, and the stabilization of a fractured vertebral column is an emergency situation so that no further damage to the spinal cord can occur.

Cross pins and PMMA could have been used, however appropriately placing implants in the thoracic vertebrae is technically difficult and has the potential risk of invading the thoracic or pleural spaces or causing iatrogenic damage to vital neurovascular structures (2, 6). Additionally, accurate drilling and placement of wires through the articular facets, as done in a modified segmental technique, are difficult in the upper thoracic spine (6). A recent study proposed pedicle screw implant corridors for normal canine T1-T9 vertebrae. However, these corridors that are presented are complex and technically challenging from a dorsal approach due to the nature of the anatomy in this region (15). Another recent study looked at implant corridors of the mid-thoracic spine in French Bulldogs. This report also shows that pedicle screw implant corridors in the mid-thoracic spine are complex and technically challenging from a dorsal approach (16).

Historically, posterior/dorsal stabilization using segmental stabilization techniques with intramedullary pins and cerclage wire has been used. Commonly referred to as spinal stapling, this segmental technique may not provide adequate stability and should be reserved for patients weighing <10–15 kgs (6, 17). As the dog, in this case, weighed 27 kgs, this technique was not indicated, again leading the surgeons to choose a dorsal locking plate apparatus.

In larger patients, the dorsal spinous processes and dorsal laminae are of adequate size and strength to allow for the placement of screws of appropriate strength. This obviated the need for exposure of the vertebral bodies and, therefore, the potential risk of entering the thoracic or pleural spaces. This fixation method, using dorsal locking plates, proved adequate in this case where complete disruption of all three compartments of the spinal column was demonstrated.

## 4. Conclusions

In conclusion, this is the first published report on the use of CT diagnostic traction to diagnose an unstable vertebral column and the first published report of a dorsal approach for placement of dorsal SOP^®^ locking plates for stabilization of a mid-thoracic fracture/luxation. This report demonstrates that the use of CT diagnostic traction, by holding the forelimbs in extension and taping the pelvic limbs to apply traction and lift the patient to the other end of the movable table, can be used to diagnose unstable vertebral columns that may initially appear stable while mitigating radiation exposure to personnel. It also demonstrates that a dorsal approach using SOP^®^ locking plates can be used to achieve adequate stabilization of the mid-thoracic vertebral column in larger patients.

In this dog, this technique was less invasive and shorter than previous reports of spinal stabilization with ventral SOP^®^ locking plate application. Limitations of this report include the single case and its retrospective nature. Further prospective studies and biomechanical evaluations of this technique are needed to corroborate what this case demonstrated. The case report demonstrated that CT scans without, then with traction allowed clear evidence of instability. The degree of instability was not evident in the initial scan without traction. Though there was evidence of a fracture, the mid-thoracic area is supported by surrounding structures which might have provided sufficient support for recovery without surgical intervention. The CT scan with traction clearly demonstrated instability that required surgical stabilization. Potential shortcomings of this approach include the cost associated with the diagnostic and surgical procedures, the dependence on patient size, and the risk of iatrogenic damage to the spinal cord.

## Data availability statement

The original contributions presented in the study are included in the article/supplementary material, further inquiries can be directed to the corresponding author.

## Ethics statement

Ethical review and approval was not required for the study of animals in accordance with the local legislation and institutional requirements.

## Author contributions

WT, PE, RB, BP, and KK contributed to the design and conception of this report. All authors contributed to manuscript revision, read, and approved the submitted version.
